# Microenvironmental interference with intra-articular stem cell regeneration influences the onset and progression of arthritis

**DOI:** 10.3389/fgene.2024.1380696

**Published:** 2024-05-22

**Authors:** Zhuce Shao, Benlong Wang, Huanshen Gao, Shenqi Zhang

**Affiliations:** Department of Joint and Sports Medicine, Zaozhuang Municipal Hospital Affiliated to Jining Medical University, Zaozhuang, Shandong, China

**Keywords:** microenvironmental, stem cell, arthritis, MSCs, interference, regeneration

## Abstract

Studies have indicated that the preservation of joint health and the facilitation of damage recovery are predominantly contingent upon the joint’s microenvironment, including cell-cell interactions, the extracellular matrix’s composition, and the existence of local growth factors. Mesenchymal stem cells (MSCs), which possess the capacity to self-renew and specialize in many directions, respond to cues from the microenvironment, and aid in the regeneration of bone and cartilage, are crucial to this process. Changes in the microenvironment (such as an increase in inflammatory mediators or the breakdown of the extracellular matrix) in the pathological context of arthritis might interfere with stem cell activation and reduce their ability to regenerate. This paper investigates the potential role of joint microenvironmental variables in promoting or inhibiting the development of arthritis by influencing stem cells’ ability to regenerate. The present status of research on stem cell activity in the joint microenvironment is also outlined, and potential directions for developing new treatments for arthritis that make use of these intervention techniques to boost stem cell regenerative potential through altering the intra-articular environment are also investigated. This review’s objectives are to investigate these processes, offer fresh perspectives, and offer a solid scientific foundation for the creation of arthritic treatment plans in the future.

## Introduction

A degenerative condition of the joints, osteoarthritis (OA) usually results in pain, stiffness, and decreased joint function (Fingleton et al., 2015; de Rooij et al., 2016; Roos and Arden, 2016). In recent times, it has emerged as a primary source of pain and impairment among the elderly (Vos et al., 2012), mainly impairing the knee’s range of motion and other typical functions (Cao et al., 2020). With osteoarthritis (OA) affecting up to 20% of the global population, the number of patients with this condition will rise as the world’s population ages. This will unavoidably lower patients’ quality of life overall and place a significant financial burden on society’s healthcare system. In orthopaedic clinical work, cartilage lesions are fairly common. However, articular cartilage lacks intrinsic healing potential and is unable to mend itself since it is an avascular, neurogenic tissue (Curl et al., 1997). Osteoarthritis (OA) and other degenerative joint disorders are caused by increasing cartilage detachment and subchondral sclerosis, which are usually the first signs of tissue degradation (Moskowitz, 2007).

Research has shown that cartilage chondrogenic cells, also known as mesenchymal stem cells (MSCs), are highly clonal, multifunctional, and chemotactic cells that live in hyaline tissues (Pittenger et al., 1999; Prockop, 2009; Hilfiker et al., 2011). When an organism sustains damage, these stem cells can precisely locate the site of the lesion while also proliferating and differentiating as necessary to replace the destroyed tissues and finish the healing process (Dupuis et al., 2007; Quesenberry et al., 2007).

In recent years, the microenvironment has become a focus of research, and it is often mentioned more frequently in tumor diseases. Of course, it also plays an important role in the occurrence and development of other diseases (Mayani et al., 1992; Kenny et al., 2007; Arneth, 2019).

Osteoclasts cause the increased subchondral bone resorption seen in the early stages of osteoarthritis (OA), and the microenvironment within the joints has a major impact on osteoclast formation and apoptosis, for example, osteoblasts, osteocytes, or activated T cells in the microenvironment, who are capable of producing osteoclast-inducing factors, which are high in the production of a factor called receptor activator of nuclear factor κB ligand (RANKL). The receptor for RANKL is RANK, and the two of them bind, the latter being the receptor expressed by osteoclast precursors, and lastly, trigger signaling cascades via adaptor proteins like Tumor Necrosis Factor Receptor Associated Factor 6 (TRAF6), which involve the pathways of nuclear factor-κB (NF-κB) and mitogen-activated protein kinase (MAPK) (Tsukasaki and Takayanagi, 2019).The results of many studies have found that osteoclasts that are highly active, which affects the histopathological changes in subchondral bone remodeling, play an important role in the development of OA, and that osteoclasts are affected by the intra-articular microenvironment, which in turn affects the microenvironment once again, resulting in a cycle that contributes to the progression of OA (Pippenger et al., 2015; Hu et al., 2021; Jiang et al., 2022).

Osteoclasts mediate increased subchondral bone resorption in early osteoarthritis. The microenvironment has an important influence on osteoclast formation, and as described in previous studies, osteoblasts, osteoclasts, and activated T cells in the intra-articular microenvironment can influence osteoclasts, as well as the function of MSCs. In addition, it is believed that the effects of the microenvironment on osteoarthritis (OA) may be mediated by stem cells, whereby the microenvironment influences stem cells, which in turn influence the ability of stem cells to repair cartilage tissue in OA.

Studies have demonstrated that the development of specific drugs can improve the animal model and the pathology of arthritis, all of which proves that the interactions between osteoclasts and the microenvironment and stem cells can be a possible avenue for the treatment of OA (Duarte, 2014). Through the secretion of immune factors, mesenchymal stem cells (MSCs) exhibit strong anti-inflammatory effects and possess immunological features that may facilitate tissue healing (Le Blanc and Mougiakakos, 2012).

Significant changes in the microenvironment will lead to changes in stem cell function, further interfering with stem cell cartilage regeneration efficacy (Berenbaum et al., 2018; Sui et al., 2019). Inflammatory variables in the joint can affect MSCs development to regulate tissue remodeling and regeneration as arthritis progresses (Shang et al., 2021). Furthermore, research has revealed that tumor necrosis factor α (TNF-ɑ) stimulates MSCs’ immunosuppressive capacity and mitigates the negative impacts of inflammatory elements in the surrounding milieu, hence facilitating tissue regeneration (Ren et al., 2008; Xu et al., 2022). Several stem/progenitor cells have been detected in bone and cartilage samples, according to the findings of several research (Zhu et al., 2010; Zhu et al., 2015; Li et al., 2020; Wang et al., 2020; He et al., 2021). Potential mechanisms and a delicate link between stem cells and the milieu were indicated by the biological targets of these cells, which included osteoclasts, dendritic cells, and macrophages (Zhu et al., 2015; Li et al., 2020; Li et al., 2021).

## The composition of intra-articular microenvironment and how microenvironment affects joint function

Extracellular matrix (ECM), various cell types, metabolic components, and certain mechanical and physical characteristics make up the intricately controlled intra-articular microenvironment. Studying joint function and disease, particularly the genesis and progression of arthritis, requires an understanding of the elements that make up the intra-articular microenvironment and how they interact.

The cells that make up the joint are mostly composed of chondrocytes, synoviocytes, stem cells, and cells that are engaged in inflammation, such T cells, neutrophils, and macrophages. While synoviocytes are in charge of producing synovial fluid, which lubricates joints, chondrocytes are crucial for preserving the integrity of cartilage tissue. Mesenchymal stem cells (MSCs), in particular, are essential for joint regeneration and repair.

A wide range of growth factors like TGF-β1 (Zhen et al., 2013; Zhen and Cao, 2014; Zhang et al., 2018a), as well as inflammatory factors like IL1β, (Fujisawa et al., 1999; Kobayashi et al., 2000; Lam et al., 2000; Kudo et al., 2003; Cao et al., 2016; Pearson et al., 2017), VEGF (Plotkin et al., 2015; Cabahug-Zuckerman et al., 2016; Dai et al., 2020) are biochemical elements in the intra-articular milieu that have a significant impact on cell activity, these elements are essential for preserving joint health, encouraging the healing of injuries, and controlling inflammation.

The mechanical and physical characteristics of the joint microenvironment—such as the tension and pressure produced by joint movement and an adequate flow of nutrients and oxygen—are essential for preserving the health of the ECM and cells. These elements work together to create a dynamic and well-balanced system that controls joint function and its capacity to heal and adapt to illness.

Numerous studies have demonstrated that TGF-β1 can inhibit stem cell activation, promote osteoclast activation that leads to bone tissue destruction, induce subchondral angiogenesis, induce hypertrophy and apoptosis of chondrocytes, and induce migration of endothelial progenitor cells and bone progenitor cells (Gerber et al., 1999; Zhen et al., 2013; Zhen and Cao, 2014; Zhang et al., 2018b; Sanchez et al., 2018; Xu et al., 2018).

Furthermore, a number of research have discovered that PGE2 and IL-6 both indirectly control chondrocytes and promote the production of osteoclasts (Liu et al., 2005; Liu et al., 2006; Ni et al., 2011). Other studies have hypothesized that TNF-α, IL-1β, and IL-6 either directly or indirectly increase the differentiation of osteoclasts (Fujisawa et al., 1999; Kobayashi et al., 2000; Lam et al., 2000; Kudo et al., 2003; Cao et al., 2016; Pearson et al., 2017).

**FIGURE 1:**
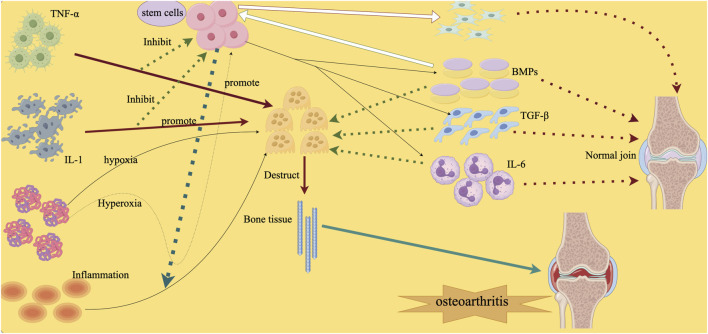
The development of arthritis is ultimately influenced by microenvironmental TNF-α and IL-1 and inflammatory factors that affect stem cells and then interfere with osteoclasts or osteoblasts. Note: Several examples of inflammatory cytokines that may be produced by inflammation include interleukin-1 (IL-1) and tumor necrosis factor- α (TNF)- α). These cytokines have the ability to activate stem cells and increase or decrease osteoclast activity, ultimately disrupting bone formation. Osteogenic growth factor (BMP) can promote the differentiation of stem cells into bone cells. The behavior of cells in joints may also be influenced by oxygen levels. Low oxygen environment can promote the development of stem cells into chondrocytes, while low oxygen environment can help activate bone cells.

Furthermore, it has been demonstrated that VEGF encourages angiogenesis and favorably influences osteoclast recruitment, which in turn influences chondrocyte activity indirectly (Lee et al., 2002; Sanchez et al., 2008).The findings of additional research have also demonstrated that VEGF and TGF-β1 stimulate angiogenesis to indirectly regulate chondrocytes (Plotkin et al., 2015; Cabahug-Zuckerman et al., 2016; Dai et al., 2020). The detailed content is shown in Figure 1.

### Influence of the intra-articular microenvironment on the function and behavior of stem cells

The maintenance of joint health and the development and progression of joint disorders are two areas where the intra-articular milieu has a significant impact on stem cell activity and behavior. Mesenchymal stem cells (MSCs), in particular, are intra-articular stem cells that are linked to arthritis pathogenesis and cartilage regeneration and repair. Stem cell destiny and activity are influenced by a variety of intra-articular microenvironmental elements, including as extracellular matrix, cellular makeup, metabolic variables, and mechanical and physical circumstances.

Osteoclasts mediate the increased subchondral bone resorption seen in early OA. The generation of osteoclasts is largely reliant on the microenvironments in which osteoblasts produce osteoblasts and osteoclasts as well as activated T cells that express molecules that induce osteoclast formation, such as receptor activators of nuclear factor κB ligand (RANKL). Tumor necrosis factor receptor-associated factor 6 (TRAF6) adapter protein activates many signaling cascades, including as the nuclear factor-κB (NF-κB) and mitogen-activated protein kinase (MAPK) pathways. Osteoclast precursors express the receptor RANK, which is bound by RANKL (Tsukasaki and Takayanagi, 2019). The activated osteoclasts will adhere to the bone and release lytic enzymes and acids that will further degrade the bone matrix. The combination of these two mechanisms may eventually lead to arthritis: Stem cell function suppression and osteoclast activation (Pippenger et al., 2015; Hu et al., 2021; Jiang et al., 2022).

Through direct cell-to-cell contact or released cytokines, intra-articular cellular components—such as chondrocytes, synoviocytes, and inflammation-associated cells—influence the proliferation, differentiation, and migration of stem cells. For instance, inflammatory cells’ cytokines may suppress stem cells’ ability to develop into cartilage while also fostering inflammation and degenerative tissue alterations.

Stem cells receive their biochemical cues and physical scaffolding from the extracellular matrix. In addition to providing structural support, extracellular matrix (ECM) constituents like collagen and proteoglycans can affect stem cell development and function by interacting with their surface receptors.

The capacity of stem cells to self-renew and differentiate is significantly impacted by inflammatory factors like IL1β and TNF-α as well as growth factors and cytokines like TGF-β and BMPs in the joint milieu. The differentiation of stem cells into certain cell lines, such chondrocytes or osteoblasts, can be encouraged or inhibited by these variables, which might impact pathological processes and joint healing.

**FIGURE 2:**
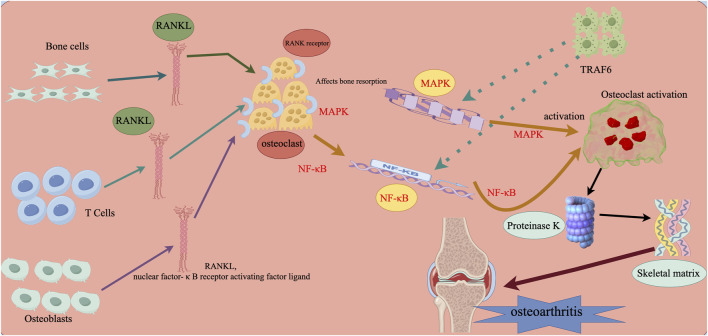
Microenvironmental influences on stem cell regeneration that further interfere with the development of arthritis. Note: RANKL (Nuclear Factor-κB Receptor Activator Ligand) is expressed by osteoblasts, osteoclasts, and activated T cells. It is an inducible factor that stimulates osteoclastogenesis in the microenvironment by attaching to RANK receptors expressed on osteoclast precursors and starting intracellular signaling pathways. Among these pathways are nuclear factor-κB (NF-κB) and mitogen-activated protein kinase (MAPK). When tumor necrosis factor receptor-associated factor 6 (TRAF6) activates the relevant signaling pathways, mature osteoclasts adhere to the bone’s surface, release proteinase K, create lysozyme and acid, and degrade the bone matrix.

Through our analysis of the influence of the microenvironment, which includes numerous factors, on stem cell regeneration and thus further interferes with the development of arthritis, we have shown as clearly as possible the process by which this mechanism occurs, as shown in Figure 2.

Stem cells in the intra-articular milieu have been shown to progress through the early, middle, and late phases of the development of arthritis. Researchers discovered signs of anabolic and catabolic metabolism in clusters of cells located in and around the articular cartilage fissures in cases of early arthritis (Lotz et al., 2010; Brack and Rando, 2012). The majority of the cells displayed Notch-1, STRO-1, and other, which are positive stem cell markers. These three stem cell markers are likewise positively stained in the central area of the arthritic cartilage (Grogan et al., 2009; Lotz et al., 2010).

### The interference of the inflammatory environment within the joint on stem cell regeneration affects arthritis

Numerous investigations have shown that the local production and release of pro-inflammatory cytokines, such as interleukin-1β (IL-1β), interleukin-6 (IL-6), and tumor necrosis factor-α (TNF-α), is a crucial component in the pathophysiology of osteoarthritis (OA) (Todhunter et al., 1996; Héraud et al., 2000; Aigner and Kim, 2002). It has been shown that nuclear factor-κB (NF-κB) is activated by TNF-α and IL-1β production. NF-κB subsequently translocates to the nucleus of the cell, where it induces inflammation, apoptosis, and the release of enzymes that degrade extracellular matrix (ECM). The expression of these factors also accelerates the extracellular matrix’s degradation and causes pain, which influences the development and progression of arthritis (Ding et al., 1998). In the intra-articular microenvironment, NF-κB promotes pro-inflammatory catabolic cytokines that in turn cause apoptosis by splitting the DNA repair enzyme poly (ADP-ribose) polymerase (PARP) and activating the pro-apoptotic enzyme caspase-3 (Shakibaei et al., 2007).

## Envisioning the future of stem cell therapy for bone and joint by interfering with stem cell regeneration in the intra-articular microenvironment

A comprehensive analysis was conducted in a study that examined the influence of the intra-articular milieu, including aged chondrocytes, on the behavior and potential for regenerative capacity of stem cells. The study underlined how crucial it is to enhance this milieu in order to raise stem cell therapy’s efficacy (Cao et al., 2019). Table 1 shows some influencing factors.

**TABLE 1:** Effects of various factors in the intra-articular microenvironment that interfere with the function of stem cells or osteoclasts.

ID	Name	Impact results
1	TGF-β1	Induction of apoptosis, chondrocyte hypertrophy, and migration of endothelium and bone progenitor cells
2	IL-6	Increases the production of osteoclasts and indirectly controls chondrocytes
3	VEGF	Influences chondrocytes indirectly while promoting angiogenesis and osteoclast recruitment
4	IL-1β	Induction of osteoclast differentiation by direct or indirect means
5	TNF-α	Induction of osteoclast differentiation by direct or indirect means
6	RANKL	Osteoclast chemotaxis and differentiation induction
7	MMP-9	Influences chondrocytes indirectly while stimulating osteoclast migration and recruitment

The use of stem cells and microenvironment regulation in the treatment of osteoarthritis, as well as the application of bioengineering technology to improve the intra-articular stem cells and microenvironment, are likely to be explored further in this field of study. Potential treatment approaches that involve altering the microenvironment of intra-articular stem cells, such as regulating inflammatory responses, enhancing the extracellular matrix’s composition, and encouraging targeted differentiation of stem cells, could be examined. The use of nanotechnology in managing the microenvironment of stem cells will also include future studies on augmenting the repair and regeneration of intra-articular stem cells by delivering growth factors, gene editing tools, and other bioactive chemicals.

In the future, with the continuous advancement of medical technology, personalized treatment will become a trend in the treatment of bone and joint diseases. By analyzing the patient’s genetic background, gene expression profile, and other information, targeted stem cell therapy plans can be implemented to maximize treatment effectiveness and prevent the occurrence of complications. Although stem cell therapy has shown great potential in the treatment of bone and joint diseases, it still faces many challenges, including uncertainty in treatment efficacy, safety considerations, and treatment costs. In the future, it is necessary to further strengthen basic research, explore the mechanisms of stem cells in bone and joint tissue regeneration, continuously improve treatment techniques and plans, in order to achieve the widespread promotion and application of stem cell therapy in clinical applications.

**FIGURE 3:**
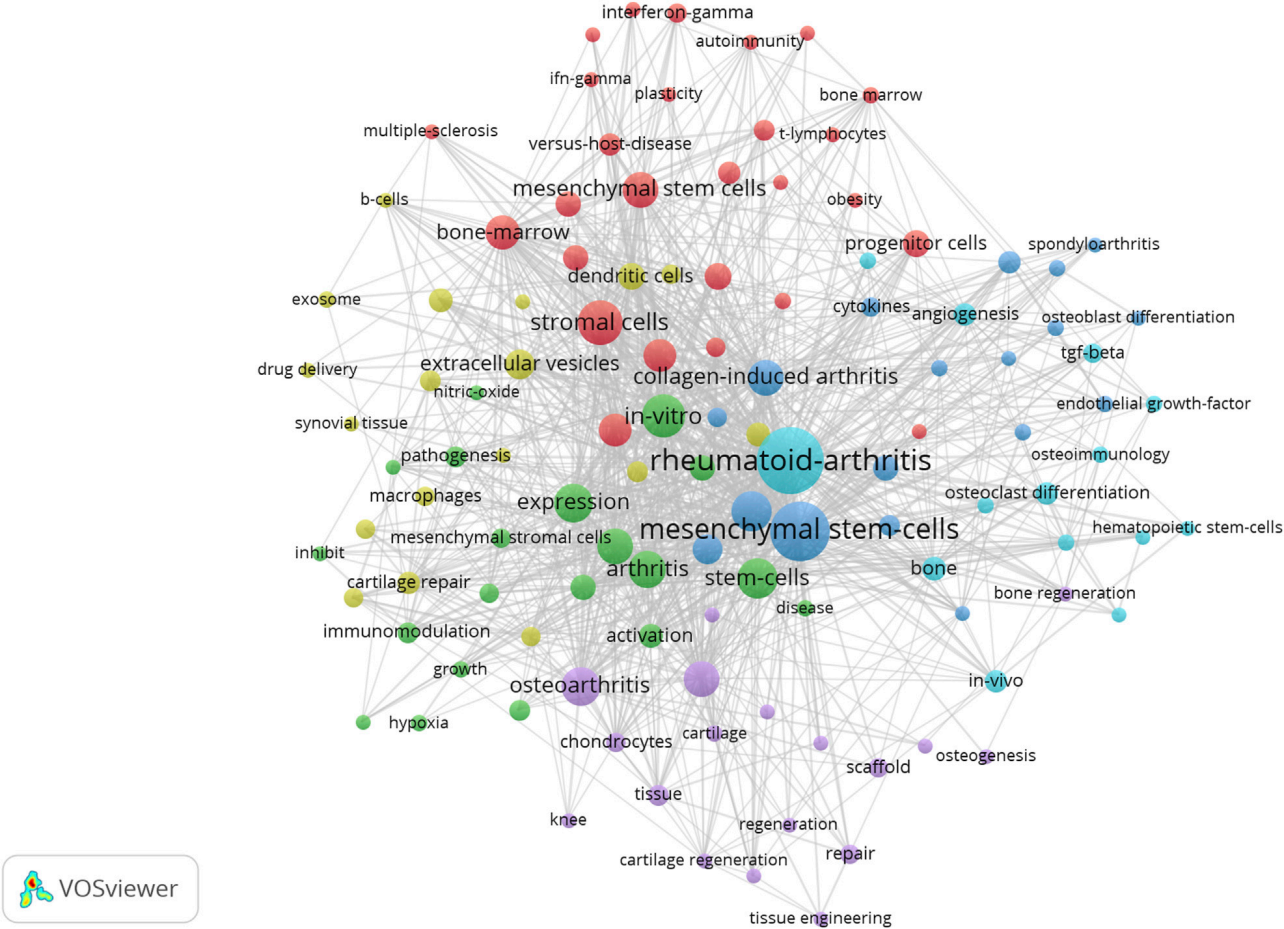
Microenvironmental disruption of intra-articular stem cell regeneration affecting arthritis Collaboration between keywords in the field of research on arthritis.

We visualize the keywords appearing in the research field of microenvironmental interference with intra-articular stem cell regeneration affecting arthritis and find the top 10 keywords with the highest frequency of keywords appearing in the research in this field, as in Table 2, from which we can find the hotspots of the research in this field and the development focuses of the attention of the researchers in the recent years, and we can intuitively find out the cooperation between the keywords appearing in the research field of microenvironmental interference with intra-articular stem cell regeneration affecting arthritis in Figure 3, where the larger the dots indicate the greater frequency of the appearances, and the number of the lines connecting the dots indicate the degree of the close relationship between the cooperating relationships.

**TABLE 2:** Microenvironmental interference with intra-articular stem cell regeneration affects the top 10 most frequently occurring keywords in the field of arthritis research.

ID	Keyword	Occurrences	Total link strength
1	Rheumatoid-arthritis	60	87
2	Mesenchymal stem-cells	48	66
3	Stromal cells	27	53
4	*In-vitro*	25	47
5	Inflammation	23	53
6	Stem-cells	23	31
7	Osteoarthritis	21	38
8	Expression	20	38
9	Arthritis	19	32
10	Collagen-induced arthritis	18	32

## Discussion

Numerous research have emphasized the impact of the body’s microenvironment on stem cells, including how it affects MSCs’ paracrine signaling (Kusuma et al., 2017), the impact of the microenvironment on stem cells’ ability to treat (Tsai et al., 2020). Stem cells have been shown in a number of studies to have an impact on arthritis, which can promote repair and regeneration of articular cartilage (Jiang et al., 2021). It can also play a role in synovial joint inflammation by itself (Bedoui et al., 2020). This suggests that the microenvironment can influence the development of arthritis and even slow down the progression of arthritic disease through its effect on stem cells.

By altering the intra-articular milieu, stem cells’ ability for regeneration can be markedly increased. For instance, by boosting certain growth factors or altering the nature of the extracellular matrix, it is feasible to promote the proliferation and differentiation of stem cells and speed up the repair of bone and cartilage (Cattaneo and McKay, 1990; Tarasenko et al., 2004; Danišovič et al., 2012; Eom et al., 2014; Qian et al., 2017). It has also been demonstrated that guided stem cell differentiation and tissue regeneration may be facilitated by using biomaterials to replicate the natural cellular milieu (Singh and Elisseeff, 2010; Griffin et al., 2015; Kuo and Rajesh, 2017).

Specific growth factors can be introduced, or the chemical and physical circumstances in the microenvironment can be altered, to encourage stem cell differentiation into chondrocytes or bone cells, which will aid in tissue regeneration and repair (Hao et al., 2017; Maisani et al., 2017; Xing et al., 2019; Zhu et al., 2021). Biomaterials, or biocompatible materials, are scaffolds that mimic the natural cellular milieu and help stem cell proliferation and differentiation (Przekora, 2019; Zhao et al., 2021a; Zhao et al., 2021b). Gene editing technology: To improve stem cells’ capacity for regrowth, the expression of certain genes is changed using CRISPR/Cas9 and other gene editing techniques (Zhang et al., 2017; Ben et al., 2018).

Stem cell research has made progress in recent years, but some obstacles remain. For example, properly regulating the activities of stem cells, including their migration, differentiation and proliferation, remains a technical challenge. There is also the fact that current research focuses on how to ensure the biocompatibility and biosafety of biomaterials, as well as how to design and prepare these materials to match the natural microenvironment. Finally, more investigations and evaluations are needed to determine the long-term effects and any adverse consequences of stem cell therapy.

Researchers remain convinced that the use of stem cell therapies to treat bone and joint disorders still holds great promise. Future advances in bioengineering and materials science, coupled with a deeper understanding of stem cells and the mechanisms that regulate their microenvironment, should pave the way for the creation of more effective therapies that will improve patient outcomes and quality of life. A major concern for researchers in this field is how to accurately regulate the activities of stem cells in the body, including their proliferation, differentiation and localization. The long-term safety and efficacy of stem cell therapy requires more research, especially the possible risks of immune rejection and tumorigenesis. Many researchers expect to try to develop standardized and reproducible stem cell therapy techniques in the near future, as well as to promote these techniques to fit clinical needs. Stem cell therapies are likely to be used more often in the future to treat bone and joint disorders. An interesting future will eventually emerge as research on stem cells and the intra-articular microenvironment deepens.

## Conclusion

In this work, we highlight the pivotal function of the intra-articular milieu in controlling stem cell renewal and its noteworthy influence on the onset and course of arthritis. The capacity of stem cells, especially mesenchymal stem cells (MSCs), to regenerate and repair injured joint tissues is dependent on how these cells interact with their surroundings. Stem cell activity can be hindered by microenvironmental changes brought on by inflammation, extracellular matrix breakdown, and disruption of cellular signaling. This can exacerbate the symptoms and course of arthritis.

Furthermore, we think that modifying the intra-articular milieu promotes stem cell-mediated regeneration, opening up exciting new therapeutic options for arthritis. Our ability to better understand these intricate relationships and create focused interventions to maximize the regenerative potential of intra-articular stem cells will open the door to novel and more successful treatments for arthritic patients, which will ultimately improve their prognosis and quality of life.
